# A Face in the Crowd: A Non-Invasive and Cost Effective Photo-Identification Methodology to Understand the Fine Scale Movement of Eastern Water Dragons

**DOI:** 10.1371/journal.pone.0096992

**Published:** 2014-05-16

**Authors:** Riana Zanarivero Gardiner, Erik Doran, Kasha Strickland, Luke Carpenter-Bundhoo, Celine Frère

**Affiliations:** 1 GeneCology Research Centre, University of the Sunshine Coast, Maroochydore DC, Queensland, Australia; 2 Centre for Ecology and Conservation, College of Life and Environmental Sciences, University of Exeter, Cornwall Campus, Falmouth, United Kingdom; 3 School of Biological Sciences, The University of Queensland, St Lucia, Queensland, Australia; Instituto de Higiene e Medicina Tropical, Portugal

## Abstract

Ectothermic vertebrates face many challenges of thermoregulation. Many species rely on behavioral thermoregulation and move within their landscape to maintain homeostasis. Understanding the fine-scale nature of this regulation through tracking techniques can provide a better understanding of the relationships between such species and their dynamic environments. The use of animal tracking and telemetry technology has allowed the extensive collection of such data which has enabled us to better understand the ways animals move within their landscape. However, such technologies do not come without certain costs: they are generally invasive, relatively expensive, can be too heavy for small sized animals and unreliable in certain habitats. This study provides a cost-effective and non-invasive method through photo-identification, to determine fine scale movements of individuals. With our methodology, we have been able to find that male eastern water dragons (*Intellagama leuseurii)* have home ranges one and a half times larger than those of females. Furthermore, we found intraspecific differences in the size of home ranges depending on the time of the day. Lastly, we found that location mostly influenced females’ home ranges, but not males and discuss why this may be so. Overall, we provide valuable information regarding the ecology of the eastern water dragon, but most importantly demonstrate that non-invasive photo-identification can be successfully applied to the study of reptiles.

## Introduction

Ectothermic vertebrates must utilize their landscape to maintain (e.g. [1], [2]). Understanding the fine-scale nature of this regulation through tracking techniques is becoming increasingly important given that environments across the world are rapidly changing. Thermoregulation is a physiological requirement for reptiles and is profoundly tied to their metabolic processes [3]; influencing their growth, rates of activity, reproductive success and body conditions [4]. Reptiles should therefore choose home ranges containing a degree of heterogeneity and complexity [4]–[6] to facilitate both physical and behavioural thermoregulation [1].

Animals concentrate their activities within a certain area defined as their home range [7]. A home range is an area familiar to the animal which usually provides all of its necessary and daily requirements such as food, retreats, mates and, in the case of reptiles, their thermal requirements [8], [9]. Home range is a dynamic phenomenon varying with sex, age, body size, reproductive status [9], season and diet [10]. For instance, when food is scarce, some animal species may expand their home ranges to find suitable food patches as seen in Bonelli’s eagles (*Aquila fasciata*) [11], wolves (*Canis lupus)* [12] and lemurs (*Lemur catta*) [13]. Many studies dedicated to understanding home ranges have shown that the size of a home range can also differ greatly between the sexes [14], [15] driven by gender-specific resource requirements [16]. For instance, the distribution of females in many mammalian species is mostly determined by the distributions of resources in the environment (reviewed in [17] as this can play a significant role in their reproductive success and their body condition [18], [19]. On the other hand, the distribution of males in most species is primarily determined by the temporal and spatial distribution of females [20], [21].

To measure the size of an individual’s home range requires the collection of spatial location data of the individual over time. The use of animal tracking and telemetry technology has allowed extensive collection of such data and has allowed us to deepen our understanding of animal movements [22], [23] and animal behaviour (e.g. [24]). Unfortunately, animal tracking and telemetry technology does not come without certain costs: they are generally invasive, relatively expensive, can be too heavy for small animals, and unreliable in certain habitats [25]. An alternative and less invasive methodology is the use of photo-identification as a means of collecting spatial information. Photo-identification can be used when individuals display unique markings. This method is commonly used in research involving mammals such as bottlenose dolphins (*Tursiops sp.*) [26], giraffes (*Giraffa camelopardalis*) [27] and kangaroos (*Macropus giganteus*) [28]. More recently, photo identification has been used to study reptiles but this usually requires animals to be caught so that specific body parts can be photographed. For instance, the Indian gliding lizard (*Draco dussumieri*) can be photo-identified using the ventral surface patterns of the patagium [29]. Similarly, the pattern of the intersections among pectoral scales were used as fingerprints in the common wall lizard (*Podarcis muralis*) and western green lizard (*Lacerta bilineata*) [30]. Our methodology aimed to be less invasive by eliminating the need to capture individuals as the handling of animals can be stressful but also time consuming.

The eastern water dragon is a protected, semi-aquatic native species with temperature-dependent sex determination. This arboreal agamid lizard (Agamidae) is found on the east coast of Australia from Queensland to Victoria [34]. Despite being an iconic Australian reptile, it is surprising how little scholarly information has been published to date about its fundamental ecology and behaviour. Being a temperature sex dependant species, eastern water dragons, like many other reptiles, are vulnerable to climate change [35] as well as ever increasing urbanisation. It is therefore important that we aim to better understand its fundamental ecology to ensure its long-term conservation.

In this study, we successfully used photo-identification to investigate gender differences in home ranges in the eastern water dragon (*Intellagama leseureuii)* and whether those differed between locations. We expected that males would have larger home ranges than females for two reasons. Firstly, males have considerably larger body sizes than females [31] and may need larger home ranges to meet their energetic requirements [32]. Secondly, males may need larger home ranges to increase their mating opportunities [31]. Finally, we also expected home range size to differ across locations as all habitats vary spatially and temporally [33].

## Materials and Methods

### Study Site and Period

**Figure 1:**
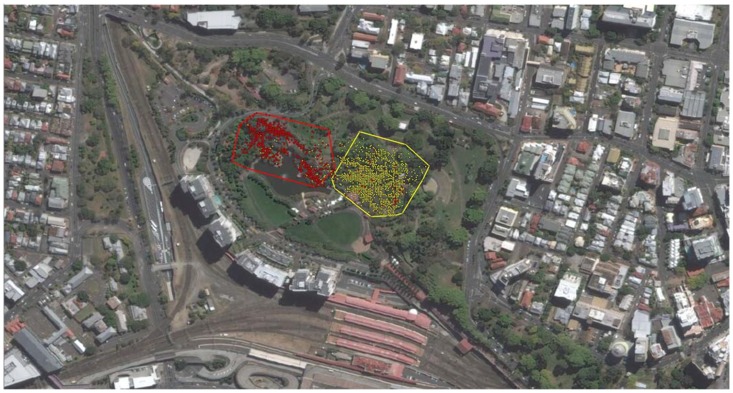
Map of study site: Roma Street Parkland, Brisbane. Areas highlighted represent Location 1 (L1) and Location 2 (L2) within the parkland. L1 contains ornamental floral arrangements, low lying bushes, playgrounds and several disconnected water features. On the other hand, L2 primarily contains native mature trees (including a rainforest); it appears more structurally complex as it also contains a large cliff, elevated bridges, and a large man-made lake.

This research was part of an on-going behavioural study of a population of eastern water dragons located at the Roma Street Parkland (the parkland), in Brisbane’s Central Business District, Australia (27° 270 4600 S, 153° 10 1100 E), which commenced in 2010. The parkland is a highly curated, man-made, subtropical garden approximately 16 hectares in size. The parkland aims to represent a diversity of habitats found across the world and has been divided into distinctive sections to represent these as such. Each distinct section of the parkland differs in size, complexity and heterogeneity of structure, vegetation and access to water. For the purpose of our investigation, we separated the parkland into 2 distinctive sections to assign general locations (see Figure 1). While we did not quantitatively measure habitat differences between the two locations, we used the following two criteria. First, there are significant floral differences between sections of the parkland. Location 1, measuring 27440.25 m^2^ in size, is a highly curated landscape and mostly contains ornamental floral arrangements, low lying bushes, playgrounds for children and several disconnected water features. On the other hand, location 2, measuring 28649.35 m^2^ in size, is significantly more structurally complex and contains a large cliff, elevated bridges, a large man-made lake and primarily contains native mature trees (including a rainforest). Second, animals seem to favour either one or the other location with very little dispersal between the two (see result section for more detail).

### Photo-identification

**Figure 2:**
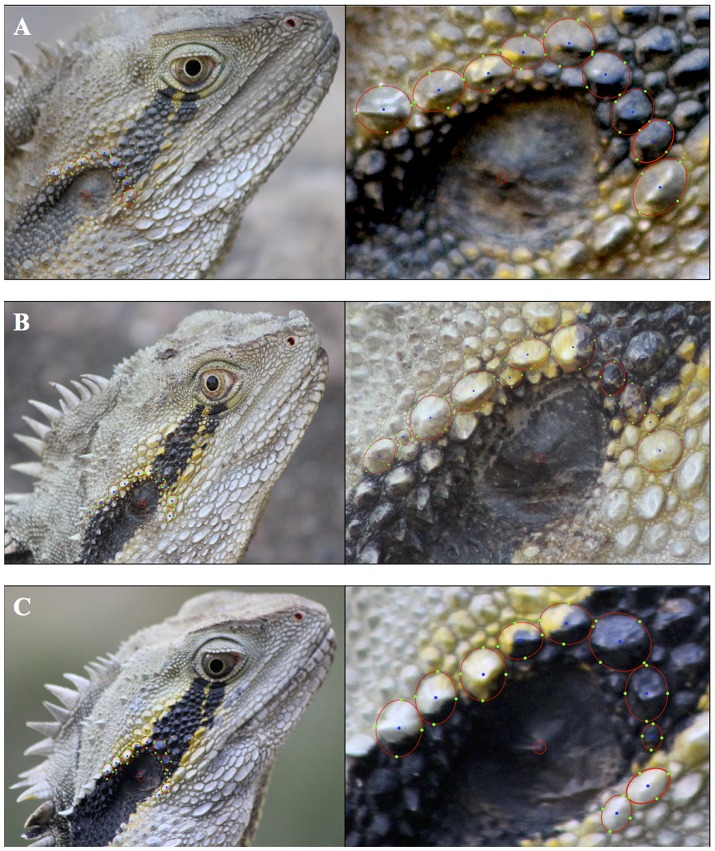
Photo-identification of lizards. Figure demonstrates how I3S Spot software was used to identify 3 different individuals (A, B and C). The ear, nose and eye of the lizard in each photograph were marked as parameters. Zooming in to photographs we marked the leading surround of the ear, which are highlighted by red ellipses characterized by 4 green dots and a central blue dot.

Photographs were collected for each individual that was sighted by an observer when walking along the parkland’s footpaths. The parkland’s footpaths are extensive and run relatively evenly across it. This allows for uniform sampling of animals throughout the parkland. We took pictures of an individual’s left and/or right profile (where possible) using a Canon EROS 600 LSD fitted with a Canon 75–300 mm lens. Similarly to Sacchi et al (29), we identified individuals using a computer-aided Interactive Individual Identification software (I^3^S Spot) created by Van Tienhoven et al. [36] (see: http://www.reijns.com/i3s/about/I3S_Spot.html) originally used to identify sand tiger sharks (*Carcharias taurus)*. Photographs were sorted into a photo library and each individual was assigned a name against a “fingerprint” or template picture reference. The ear, nose and eye of the lizard in each photograph were marked as key parameters; 7–12 scales forming the leading surround of the ear (which are unique to each individual) were then marked to reproduce their elliptical shape and size and thus produce a unique fingerprint (See Figure 2). The software ran a comparison of the fingerprint with our photo library and provided an output of the 50 closest matches. We then visually compared matches to confirm the identification or to establish the recognition of a new individual. Whilst there remains a potential for human error in this kind of visual identification, we tried to limit this by confirming any unsure or unidentified individuals with at least one other author before adding the individual to either the photo library or database. Photographs of individuals that could not be identified due to poor quality were discarded. The quality of photographs was rated according to the number of scales surrounding the ear that were clearly visible. A photograph was rated high quality if more than five scales were clearly visible and conversely as low quality if it did not.

### Home Range Size Calculations

Once a photograph was taken, we then recorded each individual’s GPS coordinates using a GARMIN eTrex10 GPS device. Sex was assigned based on morphological differences. Males are much larger in size and have a red ventral coloration. Females are much smaller in size, lack red ventral coloration and are characterised by a rounder head shape. When a confident sex determination was not possible based on these differences (usually in the case of juvenile animals), individuals were recorded as unknown sex.

Only individuals for which we had a minimum of 10 sightings collected over 245 days were included in our analysis. We found that, for both males and females, the size of the home range stabilized at or around 10 sightings. We measured this by calculating an individual’s home range size using 5, 7, 10, 12, 15, 20, 25 and 30 of its GPS points. We repeated this exercise on 10 males and 10 females. Using a linear model, we then investigated the minimum number of sightings required so that the size of an individual’s home range no longer correlated with its number of sightings, and this established that a minimum of 10 sightings was sufficient to generate accurate estimates of an individual’s home range. Moreover, in all analyses including home ranges, we included number of sightings as a fixed effect and found it was never significant.

We conducted two home range analyses. First, we investigated whether an individual’s sex and primary location influences the size of its home range. To do so, we calculated home ranges for a total of 114 individuals (males n = 50 and females n = 64) with a minimum of 10 sightings. Secondly, we investigated whether individual eastern water dragons change the size of their home range depending on the time of day (mornings: 0700–1000 hrs and afternoons: 1300–1600 hrs). To do so, we calculated home ranges for a total of 54 individuals (males n = 24 and females n  = 30) for which we had a minimum of 10 sightings for each time period (am – pm).

The size of the home range was calculated using kernel utilisation distribution methods in the adehabitatHR package [37] in R version 2.15.2. We calculated home range size at a 90% isopleth and visually estimated the smoothing bandwith (h  = 7). While different bandwidth selectors are available, such as the least cross validation and asymptotic approximation [38], [39], the most effective bandwidth selection method is by visual assessment [40]. All analyses were conducted using minimum convex polygons (MCPs) and local convex hull (LoCoH), and results showed that the patterns of significance and non-significance were the same as the kernel utilisation distribution. We therefore only show results from the kernel utilisation distribution.

To investigate whether an individual’s sex or general location influenced the size of their home range, we used a linear mixed model with individuals added as a random effect using the lme package [41] in R version 2.15.2. Prior to analyses, the homogeneity of variance and normal distribution of each variable was visually assessed by plotting residuals against fitted values. Due to a heavily right-skewed distribution, home ranges were log-transformed. Lastly, we used the related samples Wilcoxon signed rank test in SPSS to test whether individual eastern water dragons change the size of their home range depending on the time of day (mornings: 0700–1000 hrs and afternoons: 1300–1600 hrs).

## Results

### Photo-identification

Using the I^3^S Spot software, we identified a total of 586 individual animals. The density of animals varied between locations. Location 1 had the larger population with 349 individuals (males  = 172, females = 171, unknown = 6) resulting in a density of 0.0127 animals per m^2^. Location 2, on the other hand, contained 237 individuals (males = 95, females = 129, unknown = 13) resulting in a density of 0.00827 animals per m^2^. I^3^S Spot returned the correct identification within the first five matches 83.3% of searches, and within the first ten matches in 90.5% of searches. Lastly, we did not find that the quality of the photograph influenced the I^3^S Spot scoring (Wilcoxon sign rank test: n  = 304, P  = 0.08251).

### Home Range Size Variation

**Figure 3:**
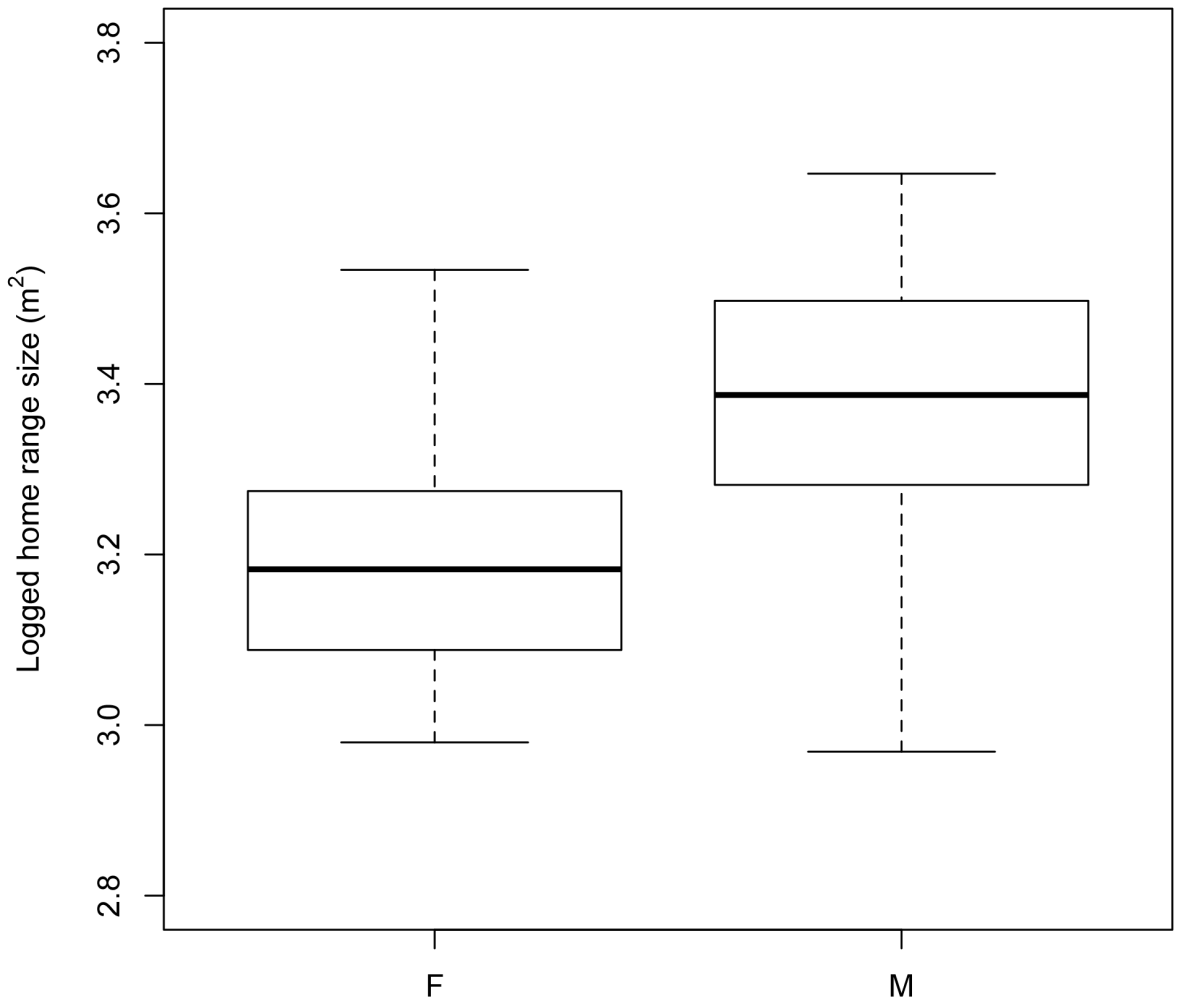
Home range size variations between sexes. Box and whisker plot showing the means, quartile ranges and medians of logged home range size (m^2^) for both females (F) and males (M). The box edges show the 25^th^ and 75^th^ percentile and the whiskers at the 10^th^ and 90^th^ percentile.

We found that the size of home ranges differed significantly between the sexes (test: LM: F _1,112_ = 36.35, P≤0.001). Males had significantly larger home range sizes than females (See Figure 3). On average the size of a male’s home range was 2464 m^2^ (SD = ±808.9), whilst a female’s home range size was approximately 1860 m^2^ (SD = ±579.9).

We found that males altered their fine scale movements between mornings and afternoons. Males had significantly larger home ranges in the mornings than in the afternoons (Related-samples Wilcoxon sign rank test: n  = 24, test statistic  = 60, S.E.  = 35, Standardized test statistic = −2.571, P  = 0.01). Females also showed fine scale movement between mornings and afternoons, however, in comparison to males, these changes were influenced by their location. Females in location 1 (park characterised by floral displays and small bushes) had larger home ranges in the mornings than in the afternoon (Related-samples Wilcoxon sign rank test: n  = 19, test statistic  = 30, S.E.  = 24.8, Standardized test statistic = −2.616, P  = 0.009). Females in location 2 had smaller home ranges in the mornings than in the afternoon (Related-samples Wilcoxon sign rank test: n  = 11, test statistic  = 64, S.E.  = 11.24, Standardized test statistic  = 2.756, P  = 0.0006). We also found that females in location 1 had significantly larger home ranges in the mornings than females in location 2 (Location 1 Mean ± SD  = 1832.3±671.8; Location 2 Mean ± SD  = 1207.3±261.1; ANOVA: F  = 8.65, df  = 29, P  = 0.006). Lastly, only 10% of individuals overlap between the 2 locations.

## Discussion

### Summary

Here, we’ve shown that the use of a non-invasive photo-identification methodology, commonly used in mammalian research, can successfully be applied to the study of the behavioural ecology of the eastern water dragon. Using this methodology, we were able to identify that a total of 586 individual eastern water dragons inhabit the Roma Street Parkland all year round. We also found that eastern water dragons exhibit intraspecific differences in the overall size of their home ranges, as well as daily variation in fine scale movements across two different locations within the parkland.

### Home Range Size Variations

We found gender differences in the sizes of home ranges. On average, males have home ranges approximately one and a half times larger than those of females. Such differences may be attributed to the apparent sexual dimorphism. Eastern water dragons follow Rensch’s rule of sexual dimorphism; males are hyperallometric, sporting larger body sizes than females [42]. Variations in home range size in sexually dimorphic species have been reported across vertebrate species such as wallabies (*Onychogalea fraenata*) [43], black footed ferrets (*Mustela nigripes*) [44], as well as in various species of lizards [6], [19], [45].

Male and female animals place varying priority over resources therefore we expect this to be reflected in the size of their home range. For instance males in many species usually compete for access to females whilst the establishment of home ranges by females may be linked to the availability and distribution of resources to optimise their reproductive success (e.g. [18], [19]). For example, Schradin et al. [46] found that female striped mice (*Rhabdomys pumilio*) established home ranges in relation to food availability and shelter. Similar findings have been found in large herbivores such as female elk (*Cervus elaphus*) [47] and marine iguanas (*Amblyrhynchus cristatus)* [48]. On the other hand, females may also establish home ranges to increase their chances of mating, as found in female Thomas langurs (*Presbytis thomasi)* which are known to disperse from their natal group and settle in new home ranges in search of reproductive males [49]. At present, we do not know whether female eastern water dragons establish home ranges according to the presence of preferred males and/or habitat quality (e.g. suitable nesting sites, shelter, basking sites, etc.). Determining such factors would provide greater understanding and inform future conservation management plans.

The observed differences in the size of home range between the sexes of eastern water dragons in this population may also be driven by the differences in their energetic requirements. Variation in the size of home range has been linked to differing energetic requirements in relation to body size [32]. As males are considerably larger than females, they are likely to have larger metabolic requirements and therefore require larger home ranges to account for this need. For instance, male polar bears (*Ursus maritimus*) which are the larger sex, have considerably larger home ranges than females [15]. Similar findings have been reported in lizards. A review by Perry and Garland [19] has reported that when male lizards are the larger sex their home ranges are twice as large as that of females. Our findings support the hypothesis that the size of home range increases allometrically with metabolic requirements (e.g. [50]).

Male eastern water dragons in the parkland have significantly larger home ranges in the morning than in the afternoon regardless of their location. These changes may be related to thermoregulation. Behavioural thermoregulation by lizards has generally been inferred by a relatively small variation in body temperature [51]. It may be that the lower morning temperatures enable eastern water dragons to be more active. Higher activity in reptiles has often been observed when temperatures are low such as mornings and or evenings. This is the case for the Australian lizard *Tiliqua rugosa* (Scincidae) which is mostly active in the early mornings [52]. Male marine iguanas *(Amblyrhynchus cristatus)* [48], [53] have also been reported to have larger home ranges in the morning. Behavioural variation in activity is an efficient method of thermoregulation in reptiles and has previously been found in a variety of reptiles, including *Ameiva exsul* [33], alligator snapping turtles (*Macroclemys temminckii*) [54], and komodo dragons (*Varanus komodoensis*) [55]. Indeed, temporal/daily variations in microhabitat selection have been found in other Australian agamids. Melville and Schulte [56] found that eight Australian agamids varied their perch height in accordance with time of day, increasing their perch height during the middle of the day when ground temperatures were highest [56].

In contrast to males, female eastern water dragons’ daily movement is influenced by location. Females found in the region of the park characterised by floral displays and small bushes (location 1) had larger home ranges in the mornings than in the afternoon. Whilst, females in location 2, characterised by mature native trees had smaller home ranges in the mornings than in the afternoon. Such differences may be due to differences in the structure between the locations.

The two locations in our study site differ in the heterogeneity of vegetation, structure, complexity and human disturbance (See Figure 1). This would most likely result in variation in the resource availability (food, basking sites, nesting sites, etc.) and exposure to extrinsic factors (sunlight, wind, rainfall, etc.). All habitats vary spatially and temporally [33] and animals, therefore, must be able to establish and adapt the size of their home range so as to provide all of their daily necessities which will be influenced by the absence or presence of desired resources [57]. For instance and similar to female eastern water dragons in the parkland, female Sika deer (*Cervus nippon)* also alter the size of their home range depending on location due to differences in resource distribution and availability [58]. Hairy-eared dwarf lemurs (*Allocebus trichotis)* had larger home ranges in dry deciduous forests within which animals had to travel greater distances to find food and water, in comparison to areas which received more rainfall [59]. Similarly, blue tongued lizards are known to constrict their home ranges in relation to food availability and cooling properties such as shade and burrows [60]. For ectotherms, choosing appropriate microhabitats is even more important given that it also has direct consequences on their ability to thermoregulate [1].

We also found that in the mornings, females found in location 1, which has a higher population density, had significantly larger home ranges than females in location 2 (where population density is lower). These results contradict patterns found in the literature where an increase in density has generally been correlated to a decrease in the size of a home range. Box turtles, for instance, had smaller home range sizes in locations with higher density [61]. Similar findings have been reported in field voles (*Microtus agrestis*) [62] and in roe deer (*Capreolus capreolus*) [63]. An increase in density leads to increased competition for resources which may drive female eastern water dragons in location 1 to increase their morning activity and thus home range to obtain sufficient resources (e.g. food, basking sites, and cooling properties). This hypothesis, however, remains to be tested. As the dataset grows, we will be able to investigate the extent to which the home ranges of eastern water dragons vary across seasons; and should investigate seasonal variation in temperature, resources and breeding. This will provide greater insights into the ways by which eastern water dragons utilise their landscape to satisfy all of their necessary and daily requirements such as food, shelter, thermal conditions, and access to mates.

### Conclusion

We demonstrated that non-invasive photo-identification can be successfully used to study the ecology of eastern water dragons and could be applied to other reptiles with similar unique surfaces. Over a 2-year period we have collected a total of 3804 photographic sightings from which we have identified more than 586 individuals using their unique facial scales without the need to catch animals. Using this methodology, we showed that eastern water dragons have extensively populated a highly curated urban park. While eastern water dragons are believed to be abundant [34], we in fact know very little about their fundamental ecology. Such gap in knowledge may severely impede our ability to protect them in the face of increasing environmental change [35].
